# Effect of Prior Austenite Grain Size on the Hydrogen Diffusion Behavior in 30MnB5 Steel

**DOI:** 10.3390/ma19050940

**Published:** 2026-02-28

**Authors:** Hyunbin Nam, Minseok Seo, Cheolho Park

**Affiliations:** Department of Welding & Joining Science Engineering, Chosun University, Gwangju 61452, Republic of Korea; hbnam12@chosun.ac.kr (H.N.); minseok0724@chosun.ac.kr (M.S.)

**Keywords:** heat treatment, prior austenite grains, grain size, martensite, hydrogen embrittlement, slow strain rate tensile test

## Abstract

In this study, we investigated the effect of heat treatment-induced grain size on the hydrogen embrittlement (HE) resistance of 30MnB5 steel, focusing particularly on the variation in prior austenite grain (PAG) size. As the heat treatment time increased, the PAGs coarsened, leading the martensite packets, blocks, and lath sizes to also coarsen. As the microstructure became more refined, the boundary density of the packet–block–lath structure increased along with a significant increase in the low-angle grain boundary (LAGB) fraction. The microstructure refinement accelerated the initial permeation rate of hydrogen, while the high density of LAGBs and trap sites effectively suppressed its long-term diffusion/localization. The slow strain rate tensile test confirmed that the tensile strength and elongation of 30MnB5 steel in a hydrogen environment were lower than those in air, indicating HE. Furthermore, the results showed that the HE sensitivity decreased in the fine microstructure condition, as evidenced by the smaller reduction in elongation compared to the coarse microstructure. The study results will enhance the understanding of hydrogen-induced degradation in hot-stamped automotive steels and offer fundamental insights for optimizing heat treatment strategies applied to 30MnB5 steel for mitigating HE.

## 1. Introduction

In recent years, the automotive industry has expanded the use of ultra-high-strength steels to achieve weight reduction and crash safety [1,2,3]. Among ultra-high-strength steels, 30MnB5 steel, manufactured using hot stamping, has the advantage of achieving extremely high strength by forming a fully martensitic structure through press forming and rapid cooling after heating to approximately 1050 °C [4,5]. However, in ultra-high-strength steels, the hydrogen introduced during manufacturing or in the service environment can be trapped at the grain boundaries, dislocations, and defects within the steel microstructures, which might reduce fracture resistance of the steels [6,7,8]. This phenomenon is known as hydrogen embrittlement; the higher the strength of the steel, the more susceptible it becomes to failure [9,10]. Particularly, in steels with martensitic structures, grain boundaries act as major diffusion paths and trap sites for hydrogen, which can lead to cracking or reduced toughness as hydrogen rapidly penetrates the material [11,12]. Several reports on the effect of prior austenite grain (PAG) size of steels, a key microstructural factor of steels, on hydrogen diffusion and brittleness have been published [13,14,15]. However, a systematic analysis of austenite grain size control under actual process conditions and its correlation with the changes in grain boundary characteristics and hydrogen trapping behavior has not yet been comprehensively conducted [14,16,17,18]. As the grain size decreases, the steel strength improves; however, the associated increase in grain boundaries and defect densities in the steel can increase the number of hydrogen trap sites, potentially leading to increased susceptibility to hydrogen embrittlement [19,20,21].

Thus, in this study, martensitic single-phase structures with different austenite grain sizes were fabricated by varying the annealing time of hot-stamped 30MnB5 steel and its resulting hydrogen diffusion characteristics, and were comprehensively investigated along with the changes in its mechanical behavior. The grain size and grain boundary characteristics were first evaluated as the functions of the heat treatment holding time. Electrochemical hydrogen permeation tests were conducted to quantify the hydrogen permeation and trapping behaviors in the microstructure of each martensitic single-phase structure. The low strain rate tensile tests were thereafter performed to compare the fracture behavior and elongation changes in the steels in air and hydrogen atmospheres and elucidate the correlation between PAG size and hydrogen embrittlement.

## 2. Materials and Methods

### 2.1. Materials

**Figure 1:**
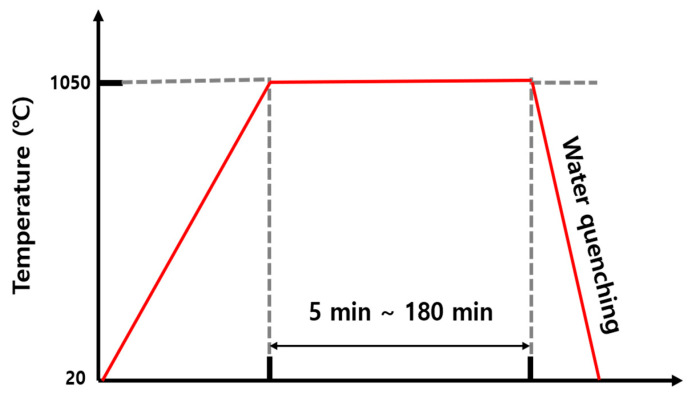
Schematic of the heat treatment cycles of 30MnB5 steel. The vertical dashed lines indicate the beginning and end of the holding stage at 1050 °C, and the horizontal dashed line represents the holding temperature.

The base metal (BM) used in this study was 30MnB5 hot-stamped steel supplied by Hyundai Steel Company, Dangjin Integrated Steelworks (Dangjin, Republic of Korea), which has attracted considerable attention owing to the increasing demand for crash safety and lightweight design in the automotive industry. It contains approximately 0.29% carbon (the chemical compositions of the hot-stamped 30MnB5 steels are listed in Table 1), which ensures high strength and toughness while providing excellent formability. The steel sheet was 1.2 mm thick, and it was subjected to additional heat treatment to obtain a microstructure suitable for hydrogen embrittlement research. The heat treatment conditions were as follows: the steel was heated to 1050 °C and then rapidly cooled via water quenching. To investigate the effect of the austenite grain size on the hydrogen diffusion behavior of the steels, the holding time at the heat treatment temperature was gradually increased from 5 to 180 min, as shown in Figure 1.

### 2.2. Microstructural Characterization

The variation in the grain size of 30MnB5 steel with heat treatment holding time was examined using optical microscopy (OM, Carl Zeiss AG, Oberkochen, Germany). Prior to using OM, the test specimen surface was mechanically polished to a final finish of 1 μm and cleaned with ethanol to remove any residual contaminants present. The grain boundaries were revealed using a Viola etchant comprising 50 mL of picric acid, 2.5 mL of hydrochloric acid (HCl), and 2 g of sodium dodecylbenzenesulfonate. After etching, microstructural images of the steel specimen were obtained under bright-field conditions to evaluate the changes in the austenite grain size. Electron backscatter diffraction (EBSD) analysis was performed to characterize the grain boundary features. EBSD enables the quantitative evaluation of grain size, shape, phase fraction, and grain boundary character distribution (GBCD) in a material, and thus EBSD is suitable for systematically elucidating the effects of heat treatment-induced microstructural changes in the material on its hydrogen diffusion and trapping behavior. The influence of the grain boundary structure of the steel on its hydrogen diffusion and trapping behavior was examined in detail by analyzing the fractions of low-angle grain boundaries (LAGBs) and high-angle grain boundaries (HAGBs) and the distribution of the coincidence site lattice boundaries.

### 2.3. Hydrogen Permeation Test

**Figure 2:**
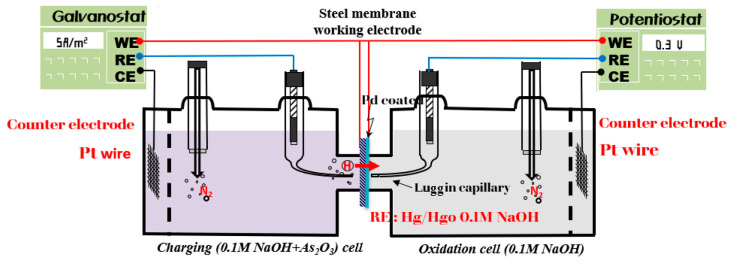
Schematic diagram of the electrochemical hydrogen permeation test setup. The blue line indicates the reference electrode, and the red line represents the working electrode.

To quantitatively analyze the hydrogen diffusion and trapping behavior of 30MnB5 steel, electrochemical hydrogen permeation tests were conducted in accordance with the ISO 17081 standard [22]. Figure 2 shows the schematic of the cell configuration used in the experiment. The tests were conducted at 300 K. The specimens were sequentially polished to 1200 # and adjusted to a thickness of 0.8 mm before being mounted at the center of a dual-cell assembly. To minimize the possibility of unintended hydrogen uptake during thin-sheet specimen preparation, all specimens were cleaned with ethanol immediately after polishing, dried with warm air, and then stored in a desiccator prior to testing.

The left cell (hydrogen charging side) was filled with a 0.1 M NaOH + As_2_O_3_ solution, while the right cell (hydrogen detection side) was purged with nitrogen gas to minimize oxygen interference, followed by filling with a 0.1 M NaOH solution. A cathodic current density of 5 A m^−2^ was applied to the charging side to generate hydrogen on the specimen surface while an anodic potential of 300 mV was applied to the detection side to oxidize the permeated hydrogen atoms. Prior to the experiment, a thin palladium film was electroplated on the specimen surface to facilitate hydrogen atom oxidation at the detection side. This pretreatment enhanced the hydrogen oxidation reaction, thereby improving the measurement sensitivity.

### 2.4. Slow Strain Rate Tensile Test

To evaluate the hydrogen embrittlement susceptibility of the steel, slow strain rate tensile (SSRT) tests were conducted in accordance with the ASTM G129 standard [23]. During an SSRT, tensile stress is applied to the test specimen at an extremely low strain rate until it fractures. The yield strength and elongation of the specimen were measured in air and under hydrogen-charged conditions. The hydrogen-charged specimens were prepared ex situ by immersing them in a 0.2 M CH_3_COONa + 0.185 M HCl solution for 6 h. After the test, the fracture surfaces of the specimen were examined using scanning electron microscopy (SEM) to quantitatively analyze the fracture modes. The analysis was used to investigate the effects of heat treatment and microstructural changes on the hydrogen embrittlement susceptibility of the steel.

## 3. Results and Discussions

### 3.1. Microstructural Changes in 30MnB5 Steel as a Function of Heat Treatment Time

**Figure 3:**
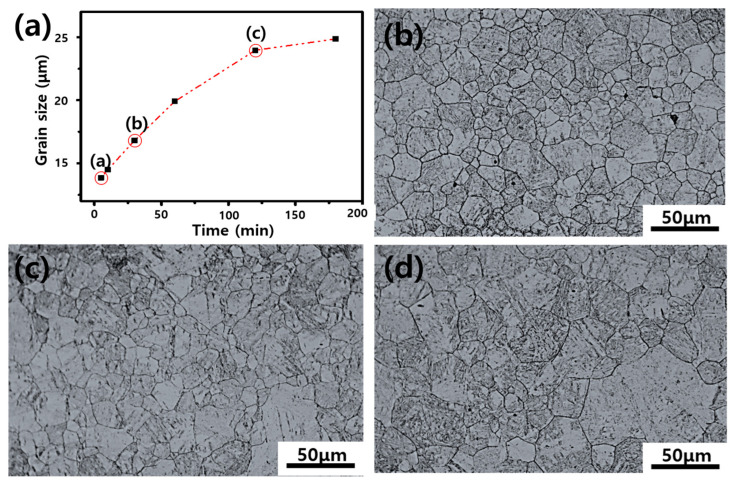
(**a**) Variation in the prior austenite grain size of 30MnB5 steel as a function of heat treatment holding time (letters a–c in the graph correspond to holding times of 5, 30, and 120 min, respectively). The red dashed line represents the grain growth trend with increasing holding time. The corresponding microstructures were obtained after holding for (**b**) 5 min, (**c**) 30 min, and (**d**) 120 min.

Figure 3 shows the PAG size of the 30MnB5 specimen as a function of the heat treatment holding time. Martensitic structures with different austenite grain sizes were obtained by heat treating 30MnB5 BM at 1050 °C for the three holding times (5, 30, and 120 min), followed by rapid water quenching. As Figure 3 shows, the OM observations revealed that the PAG size rapidly increased with increasing heat treatment holding time until it reached 30 min. Beyond 120 min, the PAG size gradually decreased. As the heat treatment time was reduced, the grains became finer and more uniformly distributed. However, longer heat treatment times resulted in coarser austenite grains, leading to an increase in the final martensitic packet size. Table 2 lists the measured grain sizes; the heat treatment holding times were set at 5, 30, and 120 min as experimental variables.

**Figure 4:**
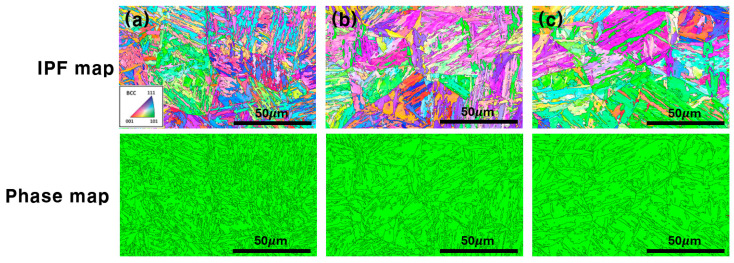
Microstructural evolution and phase analysis of each specimen as a function of heat treatment holding time: (**a**) 5, (**b**) 30, and (**c**) 120 min.

Figure 4 shows the tempered martensite structures of the specimens heat treated for 5, 30, and 120 min, observed using EBSD inverse pole figure (IPF) and phase maps. In the IPF maps, martensite structures comprising packets (MPs), blocks (MBs), and laths (MLs) were observed in all three specimens. As the heat treatment holding time increased, the PAGs became larger, and the MP and MB sizes increased. The MLs also tended to thicken. These microstructural changes are quantitatively summarized in Table 3. The grain size (G.S.) increased from 14 μm (5 min) to 24 μm (120 min), while the MP, MB, and ML sizes increased from 8.3 to 13.4 μm, 4.6 to 7.5 μm, and 1.8 to 3.0 μm, respectively, confirming systematic microstructural coarsening with prolonged holding time. When the heat treatment holding time was short, the PAG, MP, MB, and ML structures became refined, increasing their boundary density per unit area. This refinement is accompanied by a higher dislocation density, as shown in Table 3, where the dislocation density (*ρ*) decreased from 0.44 × 10^15^ m^−2^ (5 min) to 0.32 × 10^15^ m^−2^ (120 min). The behavior is closely related to hydrogen embrittlement because the dislocation density is concentrated within the MLs. Depending on the microstructural refinement degree, the boundaries can also act as trap sites. The body-centered cubic and face-centered cubic phase fractions in the heat-treated specimens for 5, 30, and 120 min were compared using EBSD phase maps. No retained austenite was detected in any of the specimens, indicating that the single-phase structure was in a fully martensitic state. Therefore, phase angle grain boundaries would be the sole variable.

**Figure 5:**
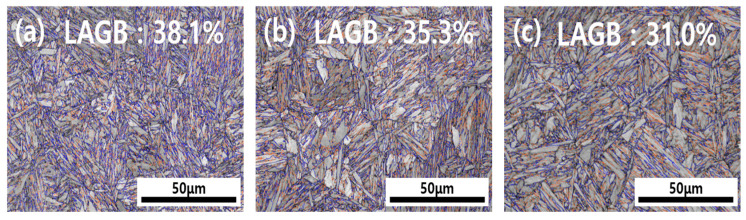
Results of the analysis of the grain boundary character distribution in the specimens for heat treatment holding times of (**a**) 5, (**b**) 30, and (**c**) 120 min. The blue lines represent high-angle grain boundaries (HAGBs, >15°), while the red lines represent low-angle grain boundaries (LAGBs, ≤15°).

Figure 5 presents the results of GBCD analysis for specimens heat treated for 5, 30, and 120 min using EBSD image quality (IQ) maps, through which the grain size and grain boundary characteristics were quantitatively evaluated. A high fraction of LAGBs was observed in the specimens heat treated for a short period (5 min); as the heat treatment duration increased, the overall grain size increased, whereas the LAGB fraction progressively decreased. The changes in the grain boundary characteristics are closely related to the hydrogen diffusion path and crack propagation resistance. The density of the lath boundaries in particular is also associated with the dislocation density and the activation conditions of the hydrogen-enhanced localized plasticity (HELP) mechanism [24,25]. This relationship arises because HAGBs are predominantly generated owing to the characteristics of the martensitic structure (packets, blocks, and laths). As the heat treatment time increased, the coarseness of the grains increased and the fraction of the HAGBs slightly increased. HAGBs possess high interfacial energy and can serve as preferential paths for intergranular cracking [26]. The LAGBs generally function as reversible trapping sites with weak binding energies. When the LAGB fraction is high, hydrogen tends to distribute more uniformly rather than being locally concentrated, which reduces the local stress concentration and consequently suppresses crack initiation. Therefore, a higher fraction of LAGBs relative to HAGBs will promote a more uniform distribution of hydrogen, reducing local hydrogen concentration and thereby mitigating hydrogen embrittlement associated with the HELP mechanism [27].

### 3.2. Hydrogen Embrittlement Susceptibility of 30MnB5 Steel with Microstructural Changes

**Figure 6:**
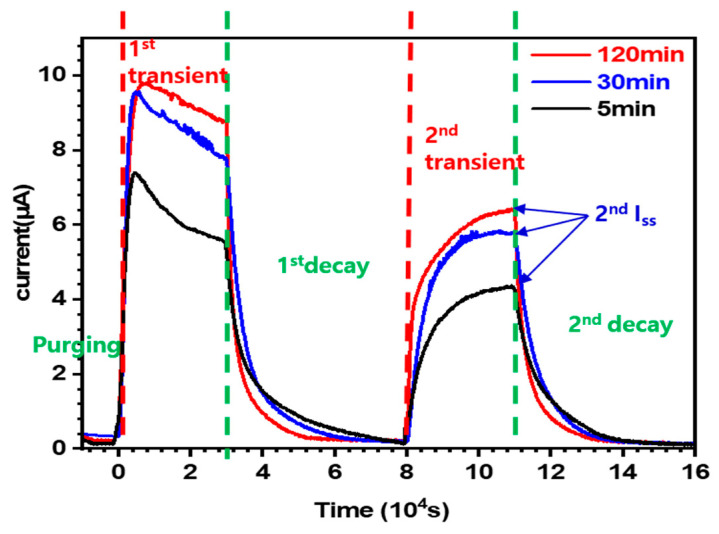
Hydrogen permeation current–time curves of specimens subjected to different heat treatment holding times (5, 30, and 120 min). The red, blue, and black solid lines correspond to holding times of 120, 30, and 5 min, respectively, while the red and green dashed lines indicate the transient and decay stages.

Figure 6 shows the current density profiles of the specimens as a function of the permeation testing time and heat treatment holding time (5, 30, and 120 min). The experimental reproducibility was verified by performing the experiment three times for each heat treatment condition. To remove hydrogen from the detection cell prior to the experiment, the cell was purged for 10,000 s at an approximate current of 300 nA. The experiment then proceeded for 160,000 s and two hydrogen permeation tests (1st and 2nd permeation), each consisting of 30,000 s of injection and 5 s of detection, were conducted during the period.

In the first permeation section (1st transient), hydrogen flowed into the specimen for the first time and most of that hydrogen was trapped at sites such as the PAGBs. Because hydrogen was repeatedly trapped and released, the current became unstable and exhibited a sharp peak, followed by a gradual decrease in the detection region (1st decay). However, in the second permeation section (2nd transient), most trap sites were already filled; therefore, only the remaining diffusible hydrogen passed through the specimen. Consequently, the current curve became much more stable. Therefore, the hydrogen diffusion behavior was analyzed using the second detection section (2nd decay). In the second decay curve, the slope was steepest in the 120 min specimen and gentlest in the 5 min specimen. The peak steady-state current (I_ss_) was highest in the 120 min specimen and lowest in the 5 min specimen.

**Figure 7:**
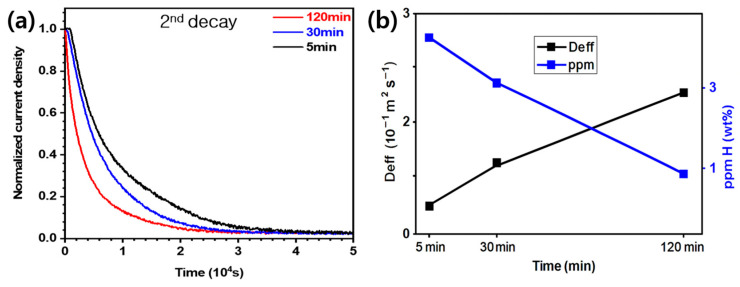
(**a**) Hydrogen permeation transient curves of the specimens subjected to different heat treatment holding times and (**b**) variations in the effective diffusivity of the specimens and their hydrogen contents, with heat treatment holding time.

Figure 7a shows the graph of the normalized current density versus time in the second decay region for the specimens heat treated for 5, 30, and 120 min. All three curves initially showed a sharp decrease, followed by gradual stabilization with increasing time. The 120 min specimen exhibited a rapid decrease in its current density at the initial stage, indicating the reduced density of reversible trap sites and consequently increased hydrogen diffusion speed. By contrast, the 5 min specimen exhibited a slow initial diffusion rate, reflecting the presence of highly dense microstructural trap sites, such as PAGBs and LAGBs, which delayed the hydrogen release. Thus, the late-time asymptotic decay regimes of all the curves converged to a similar level, whereas the initial region distinctly reflected the effect of the heat treatment time on hydrogen trapping and diffusion.

Figure 7b shows the variation in the effective hydrogen diffusion coefficient (D_eff_), along with the corresponding diffusible hydrogen concentration (ppm H) as a function of the heat treatment holding time. The D_eff_ values monotonically increased as the heat treatment time increased, indicating facilitated hydrogen diffusion due to the progressive reduction in reversible trap sites, such as PAGBs and LAGBs. By contrast, the diffusible hydrogen concentration significantly decreased with increasing heat treatment holding time. This inverse relationship demonstrated that shorter heat treatment times promoted a higher density of microstructural traps capable of holding hydrogen, whereas prolonged heat treatment reduced the overall trapping density, thereby increasing the amount of the hydrogen released.

**Figure 8:**
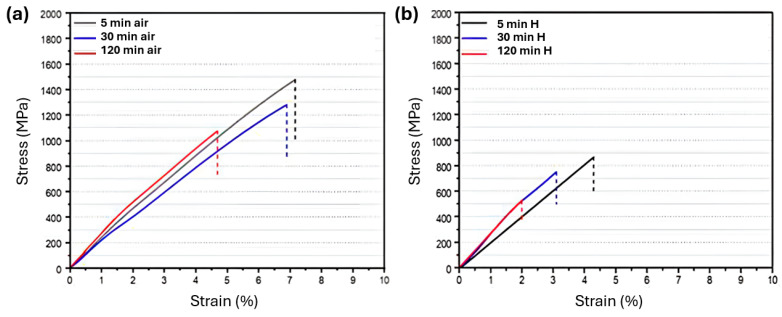
Engineering stress–strain curves of specimens subjected to different heat treatment holding times (5, 30, and 120 min): (**a**) air condition and (**b**) hydrogen-charged condition. The dashed vertical lines indicate the fracture strain of each specimen.

Figure 8 shows the engineering stress–strain curves of the specimens with and without hydrogen charging for each heat treatment holding time. The tensile properties of the uncharged specimens showed that the 5 min specimen exhibited the highest tensile strength (TS) and elongation (EL) and that the TS and EL gradually decreased with increasing heat treatment holding time. This behavior could be related to the refinement of the martensitic microstructure, which typically enhances the strength and contributes to improved ductility compared with coarser martensite. In the charged specimens, a significant decrease in both the TS and EL was observed, unlike in the uncharged specimens. The reductions in TS and EL were most pronounced in the 120 min specimen. However, the 5 min specimen exhibited a low hydrogen embrittlement index because the reductions in TS and EL were relatively small despite the high internal hydrogen solubility. With increasing heat treatment time from 5 to 120 min, the D_eff_ increased from 8.00 × 10^−12^ to 2.60 × 10^−11^ m^2^/s due to the reduction in reversible trap sites such as PAGBs and LAGBs, while the ppm H simultaneously decreased from 3.32 to 1.36, as shown in Figure 7b. Despite exhibiting the lowest ppm H, the 120 min specimen showed the highest susceptibility to hydrogen embrittlement, which is attributed to locally concentrated hydrogen trap sites. The detailed tensile properties of the specimens under both air and hydrogen-charged conditions are summarized in Table 4.

**Figure 9:**
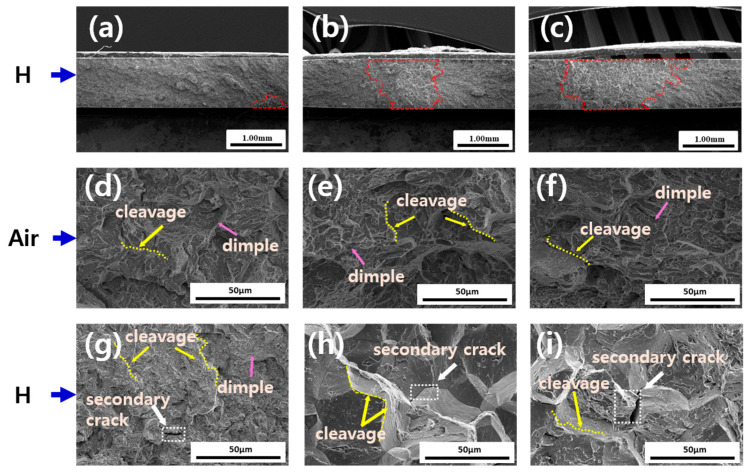
Fracture surface morphologies under different hydrogen charging conditions: (**a**–**c**) low-magnification cross-sectional views indicating damaged regions (red dotted lines); (**d**–**f**) quasi-cleavage fracture surfaces with dimples and cleavage facets (yellow dotted lines and arrows indicate cleavage regions, and purple arrows indicate dimples); and (**g**–**i**) intergranular fracture features and secondary cracks (white arrows).

Figure 9 shows the fracture surface morphologies of the specimens with and without hydrogen charging as a function of the heat treatment holding time. In all the uncharged specimens, mixed fracture patterns comprising dimples and cleavage were predominant, whereas in the hydrogen-charged specimens, mixed-mode fractures with extensive cleavage facets and secondary cracks were present. The cleavage fracture fraction markedly increased as the heat treatment holding time increased, particularly in the 120 min specimen. In the 5 min specimen, microdimple-shaped ductile fractures were more uniformly distributed than localized cleavages. This trend would have been due to the numerous trap sites within the microstructure, which effectively dispersed and delayed hydrogen, thereby suppressing local cracking. A wide cleavage region accompanied by secondary cracks was observed in the 120 min specimen, indicating pronounced brittleness. Therefore, specimens with larger PAG sizes exhibited fracture behavior that was more susceptible to hydrogen embrittlement [28]. This behavior resulted because hydrogen was allowed by the coarse-grained structure to accumulate more easily at specific interfaces or defects and the rapid crack propagation was facilitated by the small number of trap sites. In a coarse-grained structure, hydrogen can easily concentrate at the grain boundaries and defects during deformation, which promotes the formation of secondary cracks. By contrast, a fine-grained structure provides a wider grain boundary area and more trap sites, allowing hydrogen to be more uniformly dispersed, thereby reducing localized cracking.

According to the findings of this study, fine grains not only enhanced the steel strength but also activated various trap sites, which suppressed local hydrogen accumulation and improved the resistance of the steel to hydrogen embrittlement. Conversely, the reduced number of trapping sites in grains promotes localized hydrogen accumulation, increasing susceptibility to hydrogen embrittlement, whereas optimizing heat treatment conditions can mitigate this effect while maintaining the high strength and toughness of the martensitic structure. Furthermore, the suppression of hydrogen embrittlement can be maximized through grain-boundary engineering.

## 4. Conclusions

In this study, 30MnB5 steel, a hot-stamped steel, was heat treated at 1050 °C for the holding times 5, 30, and 120 min and the effects of the resulting austenite grain size changes on the martensitic microstructure and hydrogen embrittlement behavior were systematically analyzed. Based on the observations, the following conclusions were drawn:

(1) As the heat treatment holding time increased, the sizes of prior austenite grains, packets, blocks, and laths in 30MnB5 steel increased. Small prior austenite grain boundaries corresponded to a high fraction of LAGBs, which restricted crack propagation paths and improved crack propagation resistance.
(2) The 5 min specimen contained numerous trap sites that enabled rapid initial permeation while promoting long-term hydrogen dispersion and suppressing the HELP mechanism. Conversely, the 120 min specimen had fewer traps, increasing the likelihood of local hydrogen accumulation.
(3) The 5 min specimen showed excellent strength and elongation when uncharged, whereas under hydrogen charging, the 120 min specimen exhibited a pronounced drop in its strength and elongation, leading to high embrittlement indices.
(4) Under a hydrogen atmosphere, the coarse-grained specimens exhibited an increased cleavage fracture fraction and secondary cracking, indicating enhanced brittleness, whereas the fine-grained specimens showed relatively superior resistance to hydrogen embrittlement.
(5) Optimizing heat treatment and tailoring grain boundaries can effectively reduce hydrogen embrittlement while retaining martensitic strength and elongation.
(6) Future research will focus on analyzing the corrosion susceptibility of 30MnB5 steel to better understand the underlying mechanisms under different heat treatment conditions.

## Figures and Tables

**Table 1:** Chemical composition of hot-stamped 30MnB5 boron steel (wt%).

Material	C	Mn	Si	Al	Cr	N	Ni	B	Ti	Fe
30MnB5	0.29	1.39	0.21	0.03	0.21	0.005	0.038	0.004	0.036	Bal.

**Table 2:** Prior austenite grain size of the 30MnB5 specimen for different heat treatment holding times.

Holding Time (Min)	PAG Size (µm, Mean ± SD)
5	14.0 ± 1.3
10	15.0 ± 1.1
30	19.0 ± 1.3
60	20.0 ± 1.5
120	24.0 ± 1.7
180	25.0 ± 0.6

**Table 3:** Phase angle grain and martensitic substructure sizes of the 30MnB5 specimen as a function of the heat treatment holding time.

Time (min)	dMP (μm)	dMB (μm)	dML (μm)	ρ (10^15^ m^−2^)
5	8.3	4.6	1.8	0.44
30	11.5	6.4	2.6	0.39
120	13.4	7.5	3	0.32

**Table 4:** Tensile properties of 30MnB5 steel as a function of heat treatment time with and without hydrogen charging.

Time (min)	TS (MPa)	EL (%)	TS (MPa)	EL (%)
	Without Hydrogen		With Hydrogen	
5	1483	7.2	885	4.3
30	1295	6.8	748	3.2
120	1085	4.8	552	2.2

## Data Availability

The original contributions presented in this study are included in the article. Further inquiries can be directed to the corresponding author.
